# Difference in the Intestinal Microbiota between Breastfeed Infants and Infants Fed with Artificial Milk: A Systematic Review

**DOI:** 10.3390/pathogens13070533

**Published:** 2024-06-24

**Authors:** Francesco Inchingolo, Angelo Michele Inchingolo, Giulia Latini, Laura Ferrante, Elisabetta de Ruvo, Merigrazia Campanelli, Marialuisa Longo, Andrea Palermo, Alessio Danilo Inchingolo, Gianna Dipalma

**Affiliations:** 1 Interdisciplinary Department of Medicine, University of Bari “Aldo Moro”, 70124 Bari, Italy or a.inchingolo3@studenti.uniba.it (A.M.I.); or giulia.latini@uniba.it (G.L.); or laura.ferrante@uniba.it (L.F.); or elisabetta.deruvo@uniba.it (E.d.R.); mar.luis.long@gmail.com (M.L.); or a.inchingolo1@studenti.uniba.it (A.D.I.); or gianna.dipalma@uniba.it (G.D.); 2 College of Medicine and Dentistry, Birmingham B4 6BN, UK; andrea.palermo2004@libero.it

**Keywords:** gut microbiota, intestinal microbiome, neonatal milk, formula feeding, breastfeeding

## Abstract

The gut microbiota (GM) plays a crucial role in human health, particularly during the first years of life. Differences in GM between breastfed and formula (F)-fed infants may influence long-term health outcomes. This systematic review aims to compare the gut microbiota of breastfed infants with that of F-fed infants and to evaluate the clinical implications of these differences. We searched databases on Scopus, Web of Science, and Pubmed with the following keywords: “gut microbiota”, “gut microbiome”, and “neonatal milk”. The inclusion criteria were articles relating to the analysis of the intestinal microbiome of newborns in relation to the type of nutrition, clinical studies or case series, excluding reviews, meta-analyses, animal models, and in vitro studies. The screening phase ended with the selection of 13 publications for this work. Breastfed infants showed higher levels of beneficial bacteria such as Bifidobacterium and Lactobacillus, while F-fed infants had a higher prevalence of potentially pathogenic bacteria, including Clostridium difficile and Enterobacteriaceae. Infant feeding type influences the composition of oral GM significantly. Breastfeeding promotes a healthier and more diverse microbial ecosystem, which may offer protective health benefits. Future research should explore strategies to improve the GM of F-fed infants and understand the long-term health implications.

## 1. Introduction

A crucial role is played by the gut microbiome (GM) in human diseases and health, particularly in early life [1,2,3].

Trillions of microorganisms cohabit with cells inside the human organism, influencing health and quality of life [4,5,6,7]. The most important density of microorganisms is reached in the gut, where it is called the intestinal microbiome [8]. The importance of intestinal microbiome can be correlated with human development, metabolism, and behavior [9,10,11].

Microbial colonization of the intestinal tract is crucial for the maturation of the immune system in neonates. Beneficial bacteria, such as *Bifidobacterium* and *Lactobacillus*, stimulate antibody production and modulate the activity of immune cells, promoting immune tolerance and preventing autoimmune and allergic diseases. Components of breast milk, including oligosaccharides, antimicrobial proteins, and short-chain fatty acids, support the growth of beneficial bacteria and aid in the development of a balanced and protective immune system. In the intestinal system, both indigenous microorganisms and transient microorganism are present [12,13] A small number of opportunistic microorganisms can threaten intestinal health when the gut ecosystem’s balance is disrupted [14,15,16].

In breast milk, there are oligosaccharides and immunoglobulins that promote immunity and development. These promoting factors seem to protect the newborn both actively and passively, opposing intestinal diseases [17,18,19]. Factors influencing the GM in neonates include feeding and nutrition, the use of probiotics and antibiotics, gestational and postnatal age, and environmental factors.

Among environmental factors, pH, oxygen levels/redox state, the availability of water in the environment for microbial growth, and temperature can be crucial for the maturation of intestinal microbiome [20,21,22,23,24]. Other factors associated with the advance of GM of the newborn can be the type of delivery of the infant or the contact with skin during breastfeeding. GM depends on delivery mode because of the migration of the vaginal and intestinal microbiome of the mother [12,25]. Also, contamination by the skin during breastfeeding can shape GM during the first months of life of infants [26,27,28]. Although the origin of the microbiome remains unclear, an important role seems to be played by the type of nutrition, especially by components contained in mothers’ human milk [29,30,31,32,33].

The first years of life are fundamental for the development and constitution of the microbiota. It is preferable to feed the infant with breast milk for the first six months of the life of the baby because the type of feeding can condition the bacteria composition and characteristics of microbiome [13,34,35]

**Figure 1:**
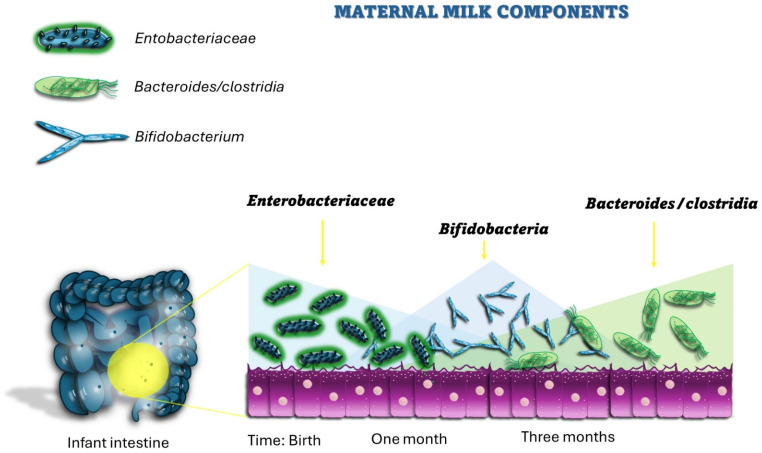
Maternal milk components can shape the development of infants’ microbiome.

Human milk given by the mother is capable of shaping the advance and the constitution of viruses, bacteria, protists, archaea and fungi, collectively termed the microbiome, that occupy infant barrier surfaces [13,36,37] (Figure 1).

**Figure 2:**
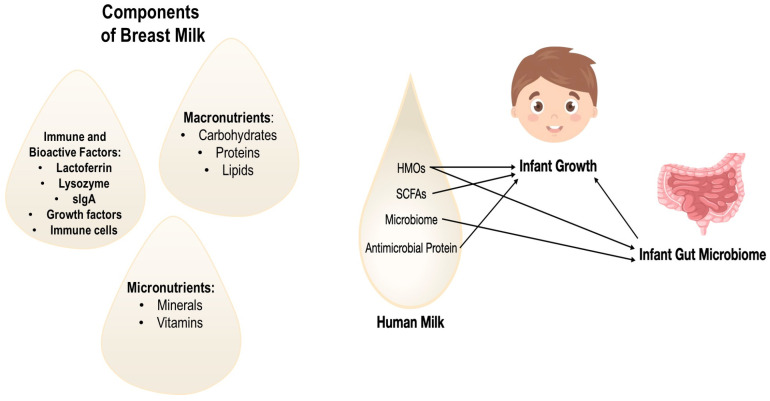
Maternal milk components can shape the development of infants’ microbiome and influence infant growth.

The effects of breast milk on shaping the development of microbiome in infants due to antimicrobial/immune-stimulatory and nutritional maternal milk proteins are particularly evident in intestinal system [38,39,40]. The human milk microbiota, human milk oligosaccharides (HMOs), short-chain fatty acids (SCFAs), and antimicrobial proteins are components of breast milk of special interest since they all affect the GM of infants, which has been linked to the body composition of babies [41,42,43]. Human milk’s SCFAs and antimicrobial proteins may also have a systemic impact on a baby’s metabolism. Additionally, there is some evidence around the impact of all these components on infant growth (Figure 2) [44].

Despite the impact of mother’s breast milk on intestinal microbiome composition, it is not fully known how human milk may affect the GM of newborn and the impact deriving from it on intestinal and general health. Through the comparison of intestinal microbiome between breastfed infants and F-fed infants, it is possible to evaluate the differences between the microbial composition and analyze the true impact of mothers’ milk on the promotion of infants’ health [5].

This systematic review aims to evaluate microbial diversity in newborns breastfed or fed with F milk from selected studies and to examine the clinical implications of the different compositions of the GM.

## 2. Materials and Methods

### 2.1. Protocol and Registration

This systematic review was conducted following the standards of the Preferred Reporting Items for Systematic Reviews and Meta-analysis (PRISMA) 2020 statement [45]. The review protocol was registered at PROSPERO under the unique number 552212.

### 2.2. Search Processing

The search period started on 10 March 2024, and the last search was carried out on 2 May 2024.

“Gut Microbiota”, “Gut Microbiome”, and “Neonatal Milk” were the search terms utilized on the databases (PubMed, Web of Science, and Scopus) to select the papers under evaluation, with the Boolean operator “AND” and “OR”. The search was restricted to just items released in English over the previous ten years (April 2014–April 2024) (Table 1).

### 2.3. Eligibility Criteria

Reviewers working in pairs conducted a comprehensive assessment of all eligible trials using the following inclusion criteria: (1) randomized controlled trials or randomized controlled clinical trials, (2) case series with more than five clinical cases, (3) studies involving human participants, (4) availability of the full text, and (5) articles published in English. The exclusion criteria were set as follows: (1) systematic or literature reviews, (2) editorials, (3) case reports, (4) case series with fewer than five cases, (5) in vitro studies, (6) studies involving animals, and (7) articles not published in English. These criteria were meticulously applied during the selection process to ensure that the studies included in this systematic review met the standards of quality and relevance.

Thos review was conducted using the PICO criteria:

- Population: term and preterm infants breastfed or artificially fed.
- Intervention: study of the microbiota of these infants.
- Comparison: analysis of the difference in the microbiome of term and preterm newborns breastfed or artificially fed.
- Outcome: main findings regarding differences in GM between breastfed and F-fed infants.

### 2.4. Data Processing

Two reviewers (G.L. and A.D.I.) independently screened the data extracted from each database using predefined inclusion and exclusion criteria. The reviewers were blinded to each other’s decisions. In a concluding meeting, both reviewers compared their results. If a reviewer deemed a paper potentially eligible, the full text was obtained and analyzed, with this process carried out independently and in duplicate. The data extracted from each eligible primary study included information such as authors and publication date, study type, study objectives, materials and methods, and results. Disagreements between reviewers regarding the article’s selection were discussed and settled.

### 2.5. Quality Assessment

The quality of the included papers was assessed by two reviewers, R.F. and E.I., using ROBINS, a tool developed to assess risk of bias in the results of non-randomized studies that compare health effects of two or more interventions. Seven points were evaluated and each was assigned a degree of bias. A third reviewer (F.I.) was consulted in the event of a disagreement until an agreement was reached. The questions in the domains evaluated in the ROBINS are the following:

- Bias due to confounding;
- Bias arising from measurement of exposure;
- Bias in the selection of participants into the study;
- Bias due to post-exposure intervention;
- Bias due to missing data;
- Bias arising from measurement of the outcome;
- Bias in the selection of the reported results.

## 3. Results

**Figure 3:**
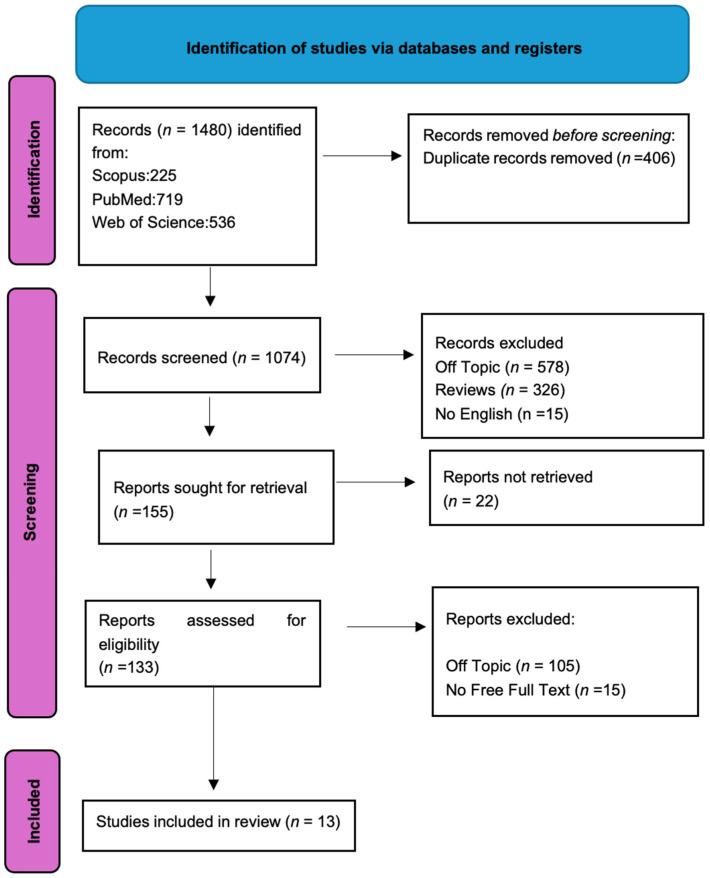
PRISMA flowchart diagram of the inclusion process. The literature search’s Preferred Reporting Items for Systematic Reviews and Meta-Analyses (PRISMA) flow diagram (Table 2).

Keyword searches of the Web of Science (536), Scopus (225), and PubMed (719) databases yielded a total of 1480 articles. After removing duplicates (406), 1074 articles remained. Out of these, 1061 were excluded for not meeting the predefined inclusion criteria. The screening process concluded with 13 publications selected for this study (Figure 3). The results of each study are presented in Table 2.

### Quality Assessment and Risk of Bias of Included Articles

**Figure 4:**
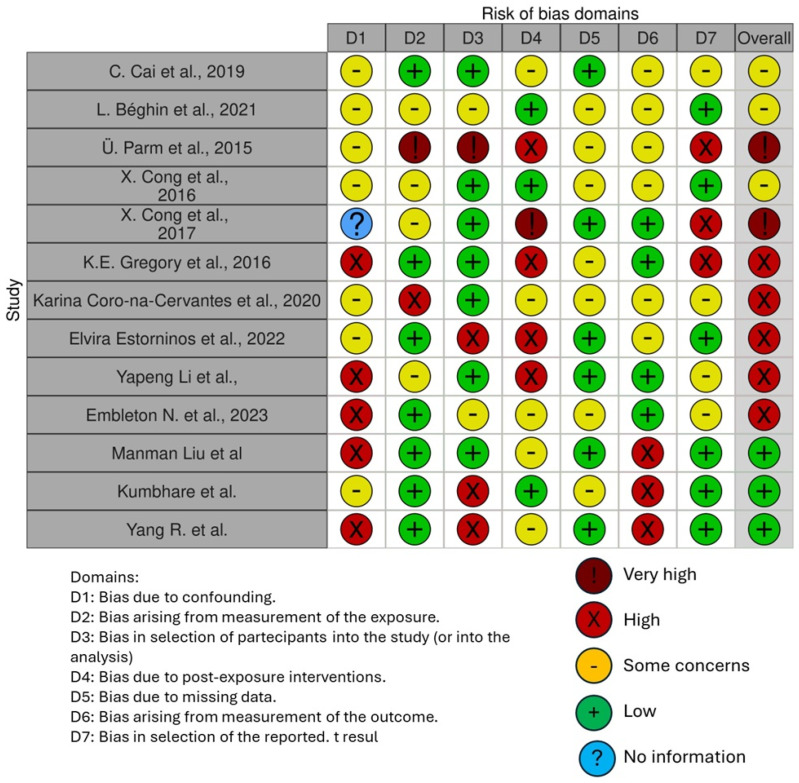
Bias assessment evaluated through ROBINS [13,17,34,46,47,48,49,50,51,52,53,54,55].

The risk of bias in the studies included is reported in Figure 4. Regarding the bias due to confounding, most studies have a high risk. The bias arising from measurement is a parameter with a low risk of bias. Many studies have a low risk of bias due to bias in the selection of participants. Bias due to post-exposure interventions cannot be calculated due to high heterogeneity. The bias due to missing data is low in many studies. Bias arising from the measurement of the outcome is low. Bias in the selection of the reported results is high in most studies. The final results show that three studies have a low risk of bias, three have a very high risk of bias, and five have a high risk of bias.

The items evaluated are as follows:

- Bias due to confounding;
- Bias arising from the measurement of exposure;
- Bias in the selection of participants into the study;
- Bias due to post-exposure intervention;
- Bias due to missing data;
- Bias arising from the measurement of the outcome;
- Bias in the selection of the reported results.

## 4. Discussion

### 4.1. Gut Microbiota of Breastfed Infants

The GM, which is essential to the health of the host, undergoes substantial alterations in infancy as a result of delivery mode, nutrition, and genetics. Breastfeeding influences the formation of an infant’s GM by supplying vital nutrients and bioactive chemicals [56,57]. Our understanding has been improved by high-throughput sequencing, which has shown that breastfed infants have unique microbial profiles with increased variety and beneficial bacteria levels. However, concerns regarding the dynamics of colonization, the role of mothers, and long-term health effects still exist [58,59,60]. The development of high-throughput sequencing tools has fundamentally changed our comprehension of the makeup and roles of the GM [61,62,63]. Research has shown that compared to their F-fed peers, breastfed infants have different microbial profiles that are marked by a higher abundance of advantageous commensal bacteria like *Bifidobacterium* and *Lactobacillus*, as well as a greater microbial variety [64,65,66,67,68].

The “Gut–Lactation Pathway” suggests that maternal gut bacteria migrate into MOM and then into the infant’s intestinal system, influencing their health. A recent study examined the microbiota of MOM and neonatal intestinal milk, highlighting variations over time and correlations between the two [69,70,71,72,73]. MOM appears to influence the infant’s GM, promoting intestinal health and reducing pathogenic bacteria. It is hypothesized that short-chain fatty acids, present in MOM, may indirectly influence the neonatal GM. Breastfeeding is considered the best way to nourish newborns, providing not only essential nutrients but also a wide range of beneficial microbes that contribute to gut health [17].

Karina Corona-Cervantes et al., in their study in 2020, explored the composition of the HM microbiota in healthy Mexican mothers and the GM of their newborns [74,75]. MOM provides a significant number of bacteria, contributing 67.7% within the first six days after giving birth. The research shows significant bacterial diversity in HM, with high abundances of Proteobacteria and Firmicutes, but with substantial differences in other taxa. It is highlighted that some bacteria in MOM can directly influence the development of the neonatal intestinal microbiota. Although the mode of delivery appears to influence the composition of the neonatal GM, a significant association between the mode of delivery and MOM microbiota has not been identified [51].

### 4.2. Gut Microbiota in F-Fed Infants

MOM is the ideal nourishment for all newborns, especially PI, as it promotes their optimal growth and development. It offers several benefits, including improved food tolerance, gut maturation, a healthy M, and enhanced immunity, reducing the risk of neonatal disease and improving neurodevelopmental outcomes. MOM contains not only essential nutrients but also bioactive components such as oligosaccharides that provide several physiological benefits, including modulation of GM and support for brain development, and infant F does not contain the oligosaccharides found in MOM, which could affect health outcomes [76]. During early childhood, the development of the GM occurs, and MOM plays a key role in supporting the colonization of GM and in the maturation of the immune system, thus influencing the future well-being of the newborn [53].

F-fed newborns have a different GM composition that is marked by fewer beneficial bacteria and less diversity due to the lack of bioactive substances in breast milk, which inhibit the growth and establishment of microorganisms [77]. Microbial profiles change because of F feeding, favoring species such as *Clostridium difficile* and *Enterobacteriaceae*, and these changes may affect long-term health consequences and metabolic pathways. The composition of the GM is also influenced by various factors and delivery mechanisms [38,78,79,80]. Prebiotics and probiotics used in F exhibit promise in modifying the composition of microorganisms and reducing related health hazards. Subsequent investigations endeavor to elucidate these interplays and formulate focused therapies to foster child well-being [81].

The American Academy of Pediatrics Section on Breastfeeding recommends six months of exclusive breastfeeding for all infants and the use of donor HM for PI when the MOM is unavailable. PI have higher nutritional requirements than TI because they miss out on the mother-to-fetus nutrient transfer that occurs later in pregnancy. The MOM of premature mothers, although richer in proteins, fats and minerals, fails to meet the nutritional requirements of the PI [82]. Embleton ND et al. showed that fortification of HM with multicomponent fortifiers increases growth and minimizes mineral deficiencies in PI, demonstrating short-term improvements in weight, length, and head circumference growth. Traditional cow’s milk fortifiers can expose the infant to cow’s milk proteins, mitigating some of the beneficial effects of HM. Recently, HM-derived fortifiers have gained popularity, with studies reporting improvements in the outcomes of PI fed fortified HM, with lower complication rates. One study compared feeding tolerance and other short-term outcomes between PI fed exclusively fortified HM and those fed MOM fortified with bovine milk fortifier or F. The results showed that infants in the exclusive HM group had greater feeding tolerance and a shorter time to achieve full enteral feeding. There were no significant differences in short-term neonatal outcomes between the groups. An exclusive HM diet is associated with improved feeding tolerance and shorter time to achieve full enteral feeding in PI [83]. Further research is needed to explore long-term effects on growth and neurodevelopmental outcomes [53].

Manman Liu et al.’s study involves 31 PI in a neonatal intensive care unit, evaluating the effects of nutrition on clinical outcomes and the correlation between intestinal microbiota and outcome. There are no significant differences in outcomes between breastfeeding and F, but there are correlations between GM and indicators such as thrombocytopenia and bilirubin. Breastfeeding appears to reduce infections and shorter hospital stays, but the composition of the GM may further influence outcomes. Breastfeeding promotes the growth of the GM, reducing the incidence of pneumonia and necrotizing enterocolitis, while F-fed PI may have a greater risk of infections and necrotizing enterocolitis. In the cohort, breastfeeding reduces the incidence of late lung infections and sepsis and accelerates the initiation of enteral feeding and discharge [84]. The correlation between GM and blood tests suggests a role of Actinobacteria and Proteobacteria in neonatal immune development [67,85,86]. Further studies are needed to confirm these findings and understand the mechanisms involved. In summary, breastfeeding reduces the risk of lung infections and late sepsis in PI, with significant implications of the gut flora in immune development [34]. The risk of necrotizing enterocolitis was lowered when human milk-based fortifier was used instead of cow’s milk-based fortifier (risk ratio 0.47, 95% CI 0.22 to 0.98) [87].

Kumbhare et al.’s study explores the impact of the type of own mother’s breastmilk fortifier and the source of HM on the composition of the GM, oxidative stress and intestinal inflammation in very-low-birthweight PI. Although the type of fortifier had little impact on GM, the source of MOM was strongly associated with its composition. Despite the differences between the fortifiers, no clear clinical impact of the human-derived fortifier was observed. A notable rise in fecal calprotectin levels was observed in infants fed this fortifier [16,88]. A significant association was also found between MOM intake and microbiota composition, along with greater weight gain and lower intestinal inflammation. The limitations of the study include the relatively small sample size and short follow-up period [66,89,90]. However, the findings emphasize the importance of maternal breastfeeding in this population of PI [54,91].

Own mother’s breastmilk contains bioactive molecules such as HM oligosaccharides that affect how the GM and peripheral nerve system grow in PI. This prospective cohort study involves 60 PI, examining changes in their GM about partial breastfeeding. Between the groups, there were no appreciable variations in several distinctive areas. Analysis of bacterial DNA showed significant differences in GM diversity between groups and over time, with a trend towards lower diversity in PI with higher MOM intake. The intake of MOM appears to positively influence the development of the intestinal microbiota, with a greater abundance of bifidobacteria. However, the study has limitations such as sample size and lack of data on maternal breast milk intake. To better comprehend the impact of breastfeeding on the GM and neurological development of PI, further research is required [90,92,93]. In conclusion, timing and breastfeeding are crucial for the development of GM during the early hospitalization of PI [55].

C. Cai et al. investigated how feeding choices influence both GM development and oxidative stress in very-low-birthweight (VLBW) PI. Using advanced sequencing techniques, fecal samples from 20 VLBW preemies were analyzed during both early and late feeding stages. The results highlighted that late stage feeding practices notably impacted both GM composition and oxidative stress levels [94]. Specifically, infants given fortified human milk or only F experienced higher oxidative stress compared to those receiving a mix of human milk (HM) and F. Diets influenced microbial diversity, and infants fed with F had fewer diverse microbes and increased levels of bacteria associated with oxidative stress. Moreover, correlation analysis uncovered links between specific bacterial types and oxidative stress markers. Notably, fortified breast milk exhibited reduced levels of bacteria associated with antioxidant defense, potentially exacerbating oxidative stress in infants [46,95,96,97].

Elvira Estorninos et al. discussed the evolution of HM-based infant F, focusing on adding bovine-milk-derived oligosaccharides to improve the F profile. Previous studies have highlighted the benefits of oligosaccharides on growth, intestinal microbiota composition and the immune response. This study examined the effects of adding oligosaccharides at a lower dose on the GM composition and intestinal immune response of F-fed versus breastfed infants. Infants fed the oligosaccharide-enriched F showed a significant increase in *Bifidobacteria*, higher levels of fecal IgA, and a better response to oral vaccines. Furthermore, they had improved gut function, suggesting that the addition of oligosaccharides to F may promote GM composition and immunity and make these more similar to those of breastfed infants [52,98].

Beneficial substances, created by specific microorganisms during fermentation, along with prebiotics, impacted the infant M. Changes in GM are specifically caused by a novel combination of bioactive substances generated by *Streptococcus thermophilus* ST065 and *Bifidobacterium breve* C50. L. Béghin et al. investigated, in their controlled, double-blind study, the comparison of F containing these substances and prebiotics to standard F and breastfed infants [99]. Over six months, various parameters were monitored, including stool secretory IgA (SIgA) concentration. The F with the blend and prebiotics notably increased SIgA levels, resembling those found in breastfed infants. Moreover, it influenced M composition, making it closer to that of breastfed infants by 4 months. Prebiotics positively impacted SIgA levels and showed normal growth and tolerance [47,100,101,102].

Ü Parm et al. aimed to explore the link between feeding methods and gut colonization in preterm infants (PI), as well as their susceptibility to late-onset sepsis (LOS) and necrotizing enterocolitis (NEC) [103,104]. Neonates receiving early enteral feeding, whether breast milk-based or F, showed increased colonization by various microorganisms compared to those on total parenteral nutrition (TPN) [105]. While enteral feeding led to higher colonization by potential pathogens, it also correlated with lower odds of LOS and mortality compared to TPN. Breast-milk-based feeding seemed to prevent colonization by certain harmful bacteria better than F or TPN. Ultimately, the study emphasizes the benefits of early enteral feeding, particularly breast milk, in reducing the risk of LOS and mortality in PI [48,106,107,108].

The composition of gut bacteria in PI evolved during the infants’ first month of life in a study conducted at the neonatal intensive care unit (NICU). The microbial DNA of stool samples was analyzed daily in stool samples from 29 PI. Certain types of bacteria, like *Clostridium* and *Bacteroides*, increased over time, while others, like *Staphylococcus* and *Haemophilus*, decreased [109,110]. This study examined changes in gut microbiome trends over time in preterm newborns. *Clostridium* and *Bacteroides* levels increased, while those of *Staphylococcus* and *Haemophilus* decreased. Daily increases in alpha diversity were noted, with significant correlations between alpha diversity and gender, feeding type, and postnatal age (*p* < 0.05–0.01). Female infants showed higher diversity and more *Clostridiates* but fewer enterobacteria than males. Infants fed mother’s own breastmilk (MBM) had higher *Lactobacillales* and *Clostridiales* levels and greater gut microbiome diversity than those fed non-MBM. The gut microbiome development in preterm infants is significantly influenced by feeding type, gender, and age [49,111,112,113].

PI were vulnerable to GM imbalances but feeding them their own mother’s breastmilk (MOM) positively influenced their gut health. The effects of different feeding approaches were monitored on PI in the NICU, and their feeding was categorized into six groups: own mother’s breastmilk, human donated milk (HDM), F, and combinations of these.

**Figure 5:**
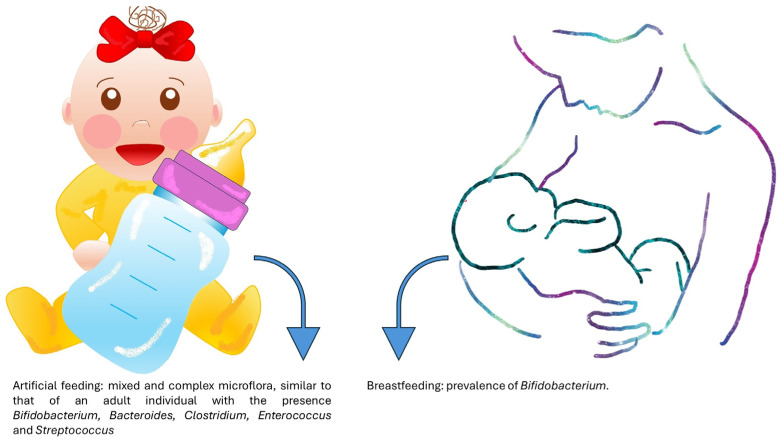
Different complex of bacterial flora in breastfeeding and artificial feeding.

Stool samples were collected daily and analyzed using DNA sequencing. PI fed own mother’s breastmilk exhibited higher levels of beneficial bacteria like *Clostridiales*, *Lactobacillales*, and *Bacillales*, whereas those fed HDM or F had more potentially harmful *Enterobacteriales*. Even after considering factors like gender, age, weight, and gestational age, PI fed own mother’s breastmilk consistently showed greater diversity in their GM over time compared to other feeding methods (Figure 5) [50,114,115,116]. When compared to infants in other feeding groups, preterm infants fed MOM (at least 70% of the entire diet) had the largest abundance of *Lactobacillales*, *Bacillales*, and *Clostridiales*, while infants fed solely HDM or formula had the highest abundance of *Enterobacteriales*. The diversity of the gut microbiome grew over time and remained consistently higher in newborns fed MOM compared to infants fed other feeding types, even after adjusting for gender, postnatal age, weight, and birth gestational age. MOM promotes the development of preterm infants’ gut microbiomes, leading to a balanced pattern of microbial community structure and enhanced microbial diversity in the early stages of life [117].

Different nutritional exposures shape the GM in PI after birth [118,119,120]. Compared to formula-fed infants, breast milk-fed newborns showed higher baseline bacterial diversity and a progressive acquisition of variety. Moreover, breast-milk-fed infants maintained a more stable GM, unlike their F-fed counterparts [121,122,123]. Supplementation with HDM helped align the GM closer to that of breast-milk-fed infants [124,125]. The crucial roles of postnatal time, birth weight, gestational age, and nutrition in molding the GM in PI have been highlighted, with breast milk offering protective benefits [126,127,128,129].

### 4.3. Inizio Modulo

This study underscores the importance of understanding how feeding choices shape the GM and its implications for VLBW preemies’ health, advocating for tailored strategies to address related health challenges. Further exploration is recommended to understand how specific bacterial by-products impact oxidative stress-related disorders in this vulnerable population [130,131,132].

## 5. Conclusions

The results of this comprehensive study show that infants given F milk and those breastfed had significantly different compositions of the GM. Compared to F-fed infants, breastfed newborns regularly have a larger microbial diversity and a higher quantity of good bacteria, such as *Lactobacillus* and *Bifidobacterium*. The distinct bioactive constituents found in human milk, including antibacterial proteins and oligosaccharides, are essential in influencing the GM and fostering intestinal well-being [117,133,134].

Conversely, babies given F have a genetically modified profile (GM profile) that is marked by a decrease in microbial diversity and an increase in the incidence of potentially harmful bacteria, including *Enterobacteriaceae* and *Clostridium difficile*. The microbial makeup of newborns given formula has changed, and this might lead to a higher risk of infections and other health problems.

The analysis also highlights how important delivery methods, in addition to the use of probiotics and prebiotics, are in influencing newborn GM. For newborns, breastfeeding remains the greatest way to nurture them since milk gives them essential nutrients and healthy bacteria. However, some of the benefits of human milk may be replicated in F which has been supplemented with certain bioactive ingredients and prebiotics.

Future studies should concentrate on comprehending the long-term health effects of these variations on GM composition and on devising methods to improve the microbial profile of F-fed infants. Further research is also required to investigate the effects of various feeding strategies on the general health and development of newborns, as well as to clarify the processes through which components of human milk influence genetically modified organisms. There are not any extensive, long-term studies that link the composition of the early microbiome to other health consequences.

In conclusion, the way that infants are fed has a significant impact on how the GM develops in their early years, with breastfeeding providing clear benefits for fostering a diversified and well-nourished microbial environment. This emphasizes how important it is for healthcare practices and policies to promote breastfeeding to maximize the health of newborns.

## Figures and Tables

**Table 1:** Database search indicators.

Article screening Strategy	Database: Scopus, Web of Science and PubMed
	Keywords: A “Gut Microbiota”; B “Gut Microbiome”; C “Neonatal Milk”
	Boolean variable: “AND” and “OR”
	Timespan: 2014–2024
	Language: English

**Table 2:** Selected literary items.

Authors	Study Design	Number of Patients	Average Age/Gender	Materials and Methods	Outcomes
C. Cai et al., 2019 [46]	Prospective cohort study	20	PI (born < 37 weeks)	Fecal samples were collected in early and late feeding stages. GM composition was evaluated through an oxidative stress marker.	GM of the infants fed the HM+ HMF diet showed an abundance of Veillonella (*p* < 0.05), which contrasted with that of the infants fed HM+ F.
L. Béghin et al., 2021 [47]	Randomized controlled trial	280	TI born between 37 and 42 weeks	The study evaluated the effects of 4 infant F in term infants (TI).	FERM/scGOS/lcFOS F brought the microbiome (M) composition closer to that of breastfed infants.
Ü. Parm et al., 2015 [48]	Multicenter study	159	PI born ≤ 32 weeks	The study evaluated the relationship between nutrition type and mucosa colonization and the development of late sepsis (LOS) and necrotizing enterocolitis (NEC) in PI.	Breast milk reduced the risk of LOS and mortality in PI.
X. Cong et al., 2016 [49]	Prospective longitudinal study	29	PI 28 weeks 0 days–32 weeks 6 days gestational age, 0–7 days old,	The study evaluated day-to-day GM patterns in PI. In total, 378 stool samples were collected daily, and Deoxyribo Nucleic Acid (DNA) extracted from stool was used to sequence the V4 region of the 16S rRNA gene region.	Infants fed their mother’s own breastmilk (MBM) had a higher diversity of GM.
X. Cong et al., 2017 [50]	Comparative study	33	PI 28 weeks 0 days–32 weeks 6 days gestational age, 0–7 days old,	The study evaluated the effect of feeding types on GM colonization of PI in NICU with the use of six types of feeding.	PI fed MOM (at least 70% of the total diet) had the highest abundance of *Clostridiales*, *Lactobacillales*, and *Bacillales* compared to other groups.
K.E. Gregory et al., 2016 [13]	Comparative study	30	PI born ≤ 32 weeks.	The PI were divided into three groups, and they were fed three types of nutrition. The GM of the groups was evaluated.	The GM is influenced by postnatal time, birth weight, gestational age, and nutrition. PI feeding with breast milk had a protective effect against gut immaturity.
Karina Corona-Cervantes et al., 2020 [51]	Descriptive cross-sectional study	67 mother-neonate pairs	age between 37 and 41 weeks	To assess the effect of human milk microbiota on the bacterial composition of the neonate’s gut in the early days, high-throughput sequencing of DNA was used.	Breast milk provides 67.7% of the bacteria in newborns within six days of birth, with significant diversity and abundance of Proteobacteria and Firmicutes. The mode of delivery influences neonatal intestinal microbiota, but not that of breast milk.
Elvira Estorninos et al., 2022 [52]	Randomized controlled trial	230 infants	21–26 days postpartum,	Both an intact cow-milk-based F and one with 7.2 g MOS/L (bovine-milk-derived oligosaccharides/L) were effective until 6 months, with gut health and immune response assessed through fecal samples.	Bovine-milk-derived oligosaccharides shift the GM and metabolic signature closer to those of human-milk-fed infants.
Yapeng Li et al. [17]	Comparative observational study	23 healthy newborns	first month of age	Samples of newborn stool and breast milk were collected. Samples collected on the day of birth (0 days) and 30 days after birth. Second-generation 16S rRNA sequencing and SCFA detection.	Construction and colonization of the intestinal microbiota in newborns. Relationship between breast milk microbiota and intestinal microbiota of newborns. Determination of short-chain fatty acids.
Embleton N. et al., 2023 [53]	Randomized clinical trial	126 PI	72 h of age	Infants were recruited from UK neonatal intensive care units and randomized to either a standard (control) or exclusive human milk diet. The control group received MOM and preterm F milk, while the intervention group received a ready-to-feed pasteurized human milk product. Data on weight gain and morbidities were collected until hospital discharge.	No impact on gut bacterial diversity in PI was found using human milk-derived F or fortifier, suggesting clinical impact is not influenced by microbiomic mechanisms.
Manman Liu et al. [34]	Retrospective study	31 infants	32 weeks	Information on duration of full enteral feeding, weight gain and postnatal infections in premature infants. Comparison of two clinical feeding methods, namely breastfeeding and F. Use of Pearson’s correlation coefficient to determine the correlation between intestinal flora and clinical outcomes.	No significant differences were found between the two feeding methods in terms of clinical indicators (duration of complete enteral feeding, weight gain and postnatal infections). Both feeding methods had no significant effect on clinical indicators in premature infants.
Kumbhare et al. [54]	Randomized clinical trial	30 PI	14 days	Comparison between two types of human milk fortifiers: Bovine-derived fortifier. Fortifier derived from human milk. Sequencing of the GM to determine microbial composition. Measurement of urinary F2-isoprostanes as a marker of oxidative stress. Measurement of fecal calprotectin as a marker of intestinal inflammation.	The source of human milk (mother vs. donor) appears to have a greater impact on the composition of the GM in PI than the type of milk fortifier (human vs. bovine).
Yang R. et al. [55]	Longitudinal observational study	60 PI	37 weeks	Basic general characteristics of premature newborns, recording of daily breast milk intake, use of probiotics and antibiotics. Collection of fecal samples at the 1st, 2nd, 3rd and 4th weeks after birth. Bioinformatic methods to analyze longitudinal intra-group variations in the structure and diversity of the intestinal microbiota, and cross-sectional differences between groups with breast milk intake >70% and ≤70%.	The development and evolution of the intestinal microbiota in premature infants during the hospital stay are continuous and non-random processes.

FERM: bioactive compounds, GM: gut microbiome, HM: human milk, HMF: human milk fortifier, lcFOS: long-chain fructo-oligosaccharides, LOS: late-onset sepsis, MOM: mother’s breastmilk, NEC: necrotizing enterocolitis, PI: preterm infants, scGOS: short-chain galactooligosaccharides, SIgA: stool secretory IgA, TI: term infants, TPN: total parenteral nutrition.

## Data Availability

Not applicable.

## Abbreviations

BM	breast milk
DNA	deoxyribo nucleic acid
F	formula
GM	gut microbiome
HDM	human donated milk
HM	human milk
HMF	human milk fortifier
M	microbiome
LOS	late-onset sepsis
MOM	own mother’s breastmilk
NEC	necrotizing enterocolitis
PI	preterm infants
SIgA	stool secretory IgA
TI	term infants
TPN	total parenteral nutrition
NICU	neonatal intensive care unit
VLBW	very low birthweight
